# Investigating the Influence of Anthropogenic Activities on Behavioral Changes of an Orb Web Spider (*Neoscona vigilans*)

**DOI:** 10.3390/insects15080609

**Published:** 2024-08-13

**Authors:** Ahmad Bilal, Abida Butt, Adeel Kazam, Shakir Ali, Isha, Young-Cheol Chang

**Affiliations:** 1 Institute of Zoology, University of the Punjab, Lahore 54590, Pakistan; ahmadbilalmalik50@gmail.com (A.B.); abidajawed.zool@pu.edu.pk (A.B.); adeelkazam07@gmail.com (A.K.); 2 Department of Zoology, Government College University, Lahore 54000, Pakistan; shakir.ali@gcu.edu.pk (S.A.); isha@gcu.edu.pk (I.); 3 Course of Chemical and Biological Engineering, Muroran Institute of Technology, Hokkaido 050-8585, Japan

**Keywords:** anthropogenic, *Neoscona vigilans*, orb web spider, road disturbance, web anomalies

## Abstract

**Simple Summary:**

Spiders are considered natural polyphagous bio-control agents of agro-ecosystems and the best indicators of environmental health. They have the ability to adapt to different environments and are, therefore, present in almost all types of terrestrial ecosystems. The behavior of spiders is influenced by different biotic and abiotic factors, such as roadside disturbance, which may affect normal web-building behavior. Spiders, *Neoscona vigilans,* were captured, body measurements were taken, and their web characteristics along the road premises were calculated. Web characteristics had a significant association with spider body characteristics and also with the distance from the road, which changed gradually as the webs were constructed closer to the road. Road disturbance had a negative impact on normal web construction as more defects were observed in the webs constructed closer to the road, while dramatically more perfect webs were observed as the distance from the road increased. We concluded that disturbances such as roadside traffic had diverse effects on spider behavior. The outcomes of this study provide insight into the role of traffic on the livelihood of spiders encountering human-influenced disturbance in the ecosystem.

**Abstract:**

Orb web spiders are common and highly diversified animals found in almost all habitats. They have remarkable plasticity against biotic and abiotic factors, making them excellent indicators of environmental health. The web creation behavior of spiders is influenced by disturbances in the environment. The aim of this research was to observe the alteration in the web-building behavior of *Neoscona vigilans* caused by human activities, specifically traffic disturbances. Spider webs were located and photographed at nighttime along the roadside, and their web characteristics were calculated. Spiders were captured from webs for their body measurements. Spider fourth leg length, carapace width, and body length had a significant association with web size and diameter, CTL, capture area, and mesh size. The quantity of trapped prey, the height of the plant, and the foliage radius increased with the distance from the road. Conversely, anchor points and web elevation from the ground dropped. The highest and lowest proportions of anomalies (modifications/defects) were recorded as holes (52.7%) in 105 webs (100%) and supernumerary (0.7%) in 55 webs (52.4%), respectively. Road disturbance had a negative influence on the spider’s behavior as the webs formed in close proximity to the road had a higher frequency of anomalies, with a gradual decrease distantly. We can gain further insight into how different environmental changes, disruptions, and pollutants lead to this imperfection in the otherwise flawless perfect structure of spider webs.

## 1. Introduction

Spiders are classified under the order Araneae, class Arachnida, sub-phylum Chelicerate, and phylum Arthropoda [1]. They are the most plentiful and varied collection of species in nearly all sorts of terrestrial ecosystems and serve as the most reliable measure of the general well-being of terrestrial communities [2]. They are regarded as polyphagous prey generalists, which means they can feed on a wide range of prey [3]. They are highly effective and significant bio-control agents for various agricultural insect pests, making them more economical, and they do not cause any greater harm to the agroecosystems, with a slight negative impact as a predator for honeybees (pollinator) [4]. This is the reason why they are included as a crucial component in the management system for biological pest control [5]. As of today, 52,011 species, 4376 genera, and 135 families worldwide have been documented. The Araneidae is the second most populous family, with a global distribution of 191 genera and 3132 species (World Spider Catalog Version 25).

The Araneidae family is widely distributed and consists of orb-weaving spiders, also known as araneids. Some of these spiders construct their webs in a consistent manner and use various types of stabilimenta (web decorations) at different stages of development to attract prey. A total of 38 spider species from the Araneidae family have been documented in Pakistan [6]. *Neoscona vigilans* is a prevalent orb-weaving spider that can be found in agricultural fields, gardens, and forest regions [7]. This creature is active during the night and hunts for prey at twilight, consuming them in the morning [8]. The spider’s web exhibits variations in slant, height from the ground, density of web lines, and magnetic orientation throughout the year, influenced by seasonal changes. However, the fundamental symmetrical structure of the web is maintained even when the spider hangs upside down. This design enables the spider to efficiently capture prey within a short period of time [9].

The presence of airborne particles in the environment is largely attributed to emissions from roadside traffic and industrial activities of human origin. The concentration of carbon monoxide (CO), ozone (O_3_), nitrogen dioxide (NO_2_), sulfur dioxide (SO_2_), nitrogen monoxide (NO), and respirable suspended particulates (RSP) is significantly elevated in the vicinity of roads due to vehicle emissions [10]. However, these pollutants decrease exponentially when measured a few meters away from the roadside [11]. PM2.5 and PM10 levels are greater in the ambient environment near roadways due to the creation of local road dust [12]. This is caused by the dry road surface, high wind speed, and the weight and speed of vehicles on the roads. The typical anatomical structure of spiders can be altered by various factors, such as growth, food availability, leg regeneration, silk production, experience, and egg production [13]. Additionally, environmental factors, such as temperature, light, humidity, gravity, wind, web support, prey abundance and quality, and interactions with other spiders of the same species, can also impact the web structure [14].

Excessive disruption in the natural environment leads to abnormalities in spider webs. They have the ability to assimilate contaminants and emissions, making them a reliable indicator of air pollution [15]. Spider webs serve as important indicators for measuring the levels of polycyclic aromatic hydrocarbons (PAHs) in roadside traffic emissions. These levels can change depending on the circumstances [16]. Mobile sources emit gases and pollutants at higher levels near roadways, resulting in increased exposure for species that spend a substantial amount of time in that specific area [17]. These substances experience both wet and dry depositions and become trapped in spider webs [18]. The presence of heavy metals in airborne particulate matter poses a significant risk to the environment and living beings due to their metabolic activity [19]. Despite the unpredictable nature of wind in the environment, spiders adapt their web structure to efficiently capture prey by keeping it under high wind pressure [20,21] through the creation of smaller webs. Wind pressure or air blow can cause an increase in the evaporation of water from sticky spirals, resulting in a decrease in the stickiness of spider webs [22]. This decrease in stickiness can then reduce the spiders’ capacity to capture prey. The behavioral reactions, specifically missed detection and false alarms, are influenced by powerful airborne vibrations originating from the surrounding environment [23].

Environmental factors contribute to the variability in the regularity and symmetry of spider webs [24]. Web variation is influenced not just by environmental conditions but also by the activity of the spider [25]. Variations in the extent of web breakage and prey capture suggest that various spider species respond differently to challenging environmental conditions [26]. Various environmental conditions influence the web-building behavior of spiders. The production of anomalies in spider webs is caused by changes in the behavior of spiders [25]. The laboratory conditions under which the webs were constructed exhibited anomalies, suggesting a deviation from the ideal structure caused by a shift in the spiders’ behavior [27]. The geometric structure of spider webs undergoes changes as spiders age, resulting in the introduction of more anomalies. These anomalies, or adjustments in the web’s structure, decrease the spider’s capacity to capture prey. This decrease in prey-catching ability is attributed to the variability in the spider’s web-building activity. There are positive relationships between the number of anomalies and certain web characteristics, such as mesh size, capture area, capture thread length, and width of the lowest half of the web [28,29,30]. The existence of anomalies indicates a flawed construction of webs. Roads are the primary factor contributing to the division and destruction of ecosystems [31]. Disturbances occurring along roadsides have a direct impact on the behavior and physiology of animals, leading to a decrease in their overall success.

Spiders’ behavior and ability to build webs are negatively affected by the presence of high traffic pressure and the pollutants it generates [32]. The objective of this research was to observe the alteration in the web-building behavior of spiders due to the influence of pollutants and disturbances in the roadside environment.

Subsequent research endeavors could examine the effects of traffic pollution on spider populations throughout extended temporal scales and evaluate the possibility of spider adaptation to traffic disturbances. This also warrants future investigations on spiders’ gene flow and dispersal due to traffic commotion, ultimately promoting conservation efforts and their coexistence in the subsequent environment.

## 2. Materials and Methods

The study site was a bustling road located at latitude N 31°53″–31°55″ and longitude E 72°28″–72°29″. This route runs through the citrus orchards of Sargodha, Punjab, Pakistan. During the period from 2000 PST to 2400 PST, we collected data, captured images by giving a black background with black sheets and highlighting webs by flashlights, and made measurements of web properties. We discovered and photographed the webs of our target species by traveling from the roadside to a distance of 400 m in the orchard. Foliage radius was measured by measuring vertical and horizontal expansion of plant leaves and branches, then taking their average of every plant where the web was located. Anchor points were extra threads other than radii and spirals. They allow the web to maintain position and attach to the plant or any other objects, which were measured accordingly. Plant height was measured with the help of trees application (forest monitoring tool). We also recorded the coordinates of each web. Each spider from every web was collected and preserved in vials containing a solution consisting of 80% alcohol and 20% glycerin. Measurements of body length, carapace breadth, and length of the 4th leg were conducted in a laboratory setting [33].

### 2.1. Spider Identification

**Figure 1:**
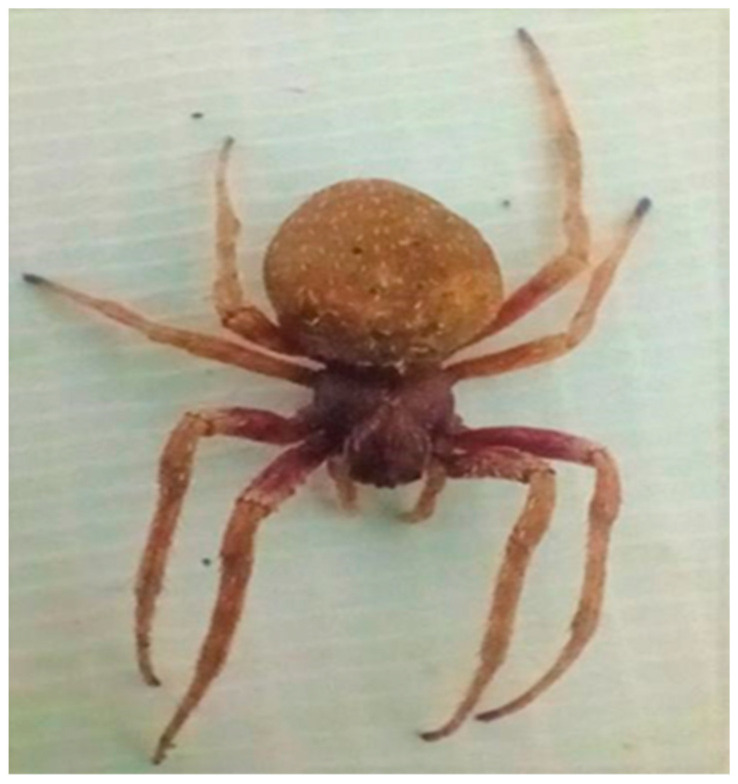
Spider’s dorsal view.

**Figure 2:**
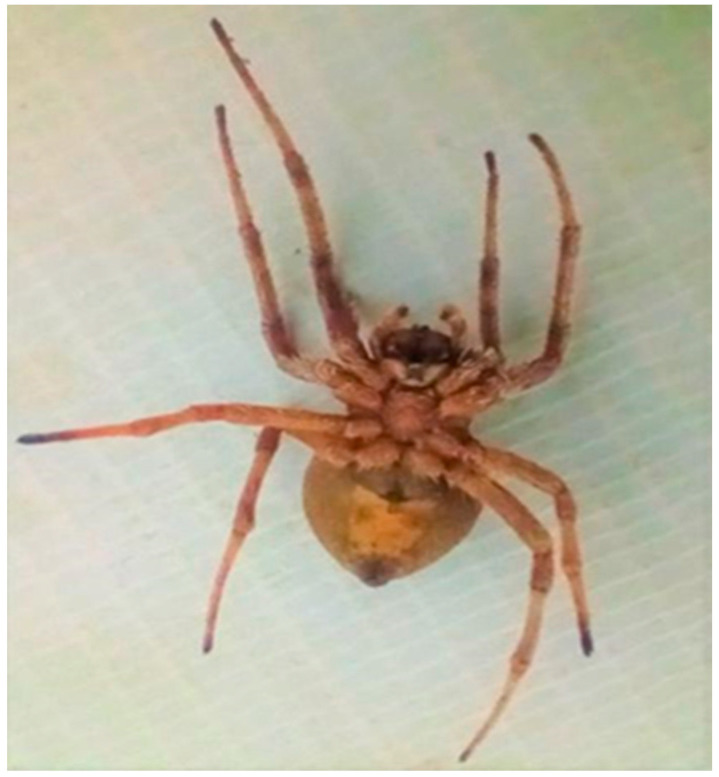
Spider’s ventral view.

The spider was recognized as *Neoscona vigilans* [34] (Figure 1 and Figure 2) using a stereomicroscope and referencing the keys and research [35]. Earlier, it was called *Neoscona rumpfi* [36].

### 2.2. Data Analysis

**Figure 3:**
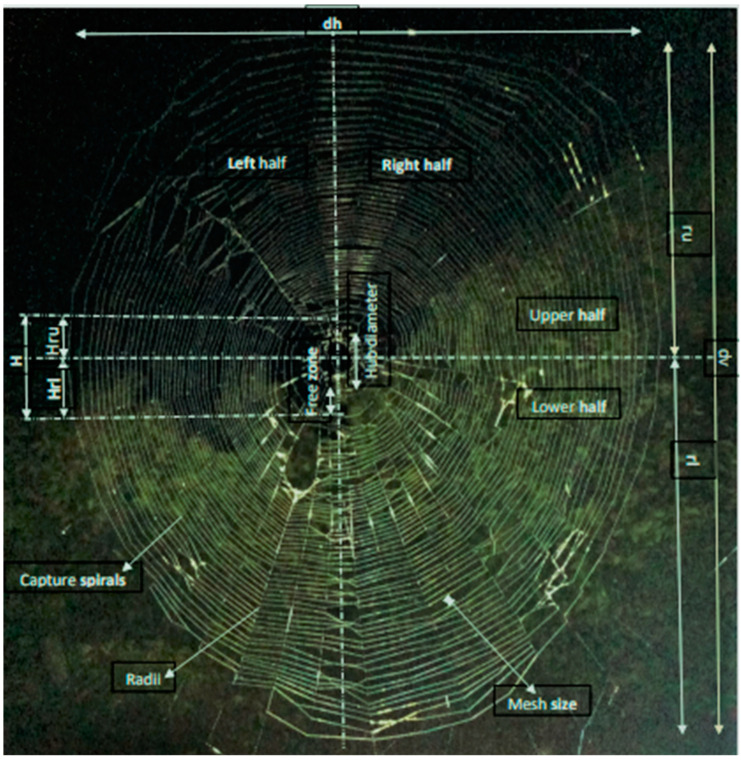
Web characteristics.

The following method was used to establish the capture area (Figure 3), which is the part of the web that the capture spirals cover [37].

$$\left[\frac{1}{2}\pi r_{au}^{2}-21\pi {\left(Hr_{u}\right)}^{2}\right]+\left[\frac{1}{2}\pi r_{al}^{2}-21\pi {\left(Hr_{l}\right)}^{2}\right]$$

$$r_{au}=\frac{{r_{u}}_{+\frac{d_{h}}{2}}}{2}\;$$

$$r_{al}=\frac{{r_{l}}_{+\frac{d_{h}}{2}}}{2}\;$$

The dimensions required are as follows: the upper radius (*r_u_*) and lower radius (*r_l_*) of the web, which include the hub and free zone; the upper hub radius (*Hr_u_*) and lower hub radius (*Hr_l_*), which also include the free zone; and the horizontal diameter of the web (*d_h_*), which includes both the hub and free zone.

A formula was used to measure the mesh size, which is the distance between the capture spirals, for each web [37].

$$\left[\frac{1}{2}\left(\frac{r_{u}-Hr_{u}}{{(s}_{u}-1)}+\frac{r_{l}-Hr_{l}}{{(s}_{l}-1)}\right)\right]$$

How many spirals make up the top half (*S_u_*) and how many make up the bottom half (*S_l_*)? The formula from [38] was used to compute capture thread length (CTL).

$$\left[\frac{\pi }{16}\times \left(N_{v}+N_{h}\right)\times (D_{ov}+D_{iv}+D_{oh}+D_{ih})\right]$$

The following aspects were considered: vertical diameter (*D_ov_*), horizontal diameter (*D_oh_*), horizontal hub diameter (*D_ih_*), vertical hub diameter (*D_iv_*), number of horizontal and vertical spirals (*N_h_*, *N_v_*).

Web size was formula-based [39].

$$\left(\frac{d_{v+}d_{h}}{2}\right)$$

The horizontal diameter of the web (*d_h_*) and the vertical diameter of the web (*d_v_*) encompass the hub and the free zone diameter.

Formulae were used to establish web asymmetry [40].

$$\left(\frac{r_{u}-r_{l}}{r_{u}+r_{l}}\right)$$

### 2.3. Web Anomalies

**Figure 4:**
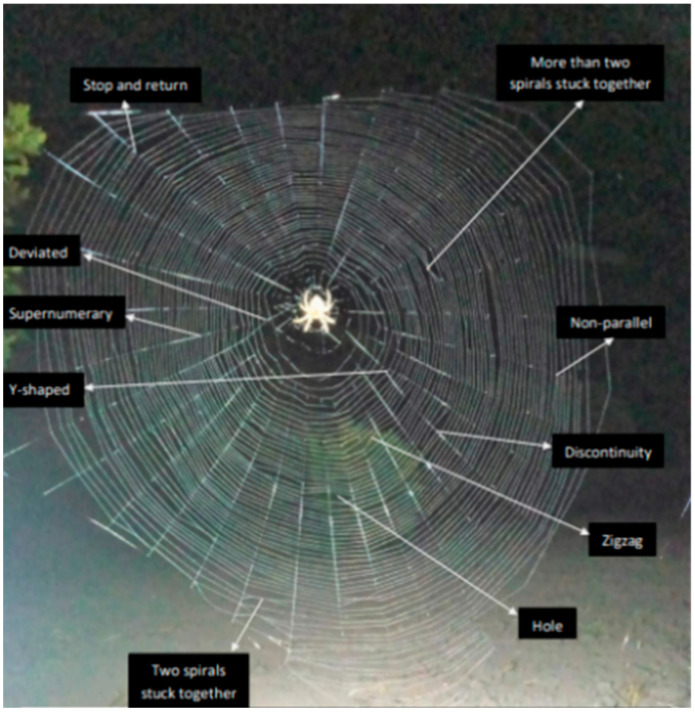
Web anomalies.

Figure 4 depicts spiral and radial anomalies that were documented [27].

Supernumerary refers to the radius of the orb that does not emanate from the center of the web.

Deviated: a radius that is not straight and is more than 5° off from a straight-line path starting from the middle of the web.

Y-shaped: a radius that starts from the center of the web and then divides into two distinct segments, each reaching the frame thread at a different place.

Stop and return: the spiral unit stops at a certain radius and starts all over again at the same radius in the same area.

Hole: at least one spiral unit is absent, and it is surrounded by at least two spiral units on each side.

Two spirals stuck together: two consecutive spiral units are fused together within a sector.

More than two spirals are stuck together: specifically, three or more spiral units are fused together within a sector.

Discontinuity: a spiral thread terminates at a radius and is bordered on both sides by two separate spiral units that are not linked to it.

Non-parallel: when two spiral units meet at a point on the radius, they form a triangle that goes into the next sector and continues on.

Zigzag: a spiral unit that changes direction in at least three successive sectors.

### 2.4. Statistical Analysis

Statistical analysis of collected data was carried out using SPSS version 21, Microsoft Excel version 2013, and ImageJ software version 1.8.0_60, UTM GEO MAP version 3.0.2. Normality of data was checked through Shapiro–Wilk test (*p* > 0.05). Through the utilization of distances measured from the road, we successfully computed the correlation between each web anomaly and the unique attributes of the spiders and their webs by using Pearson correlation. In conjunction with distance from the road, scatter plots representing each web anomaly were generated. And, using a pie chart, the proportion of each web anomaly was ascertained.

## 3. Results

### 3.1. Anomalies

**Figure 5:**
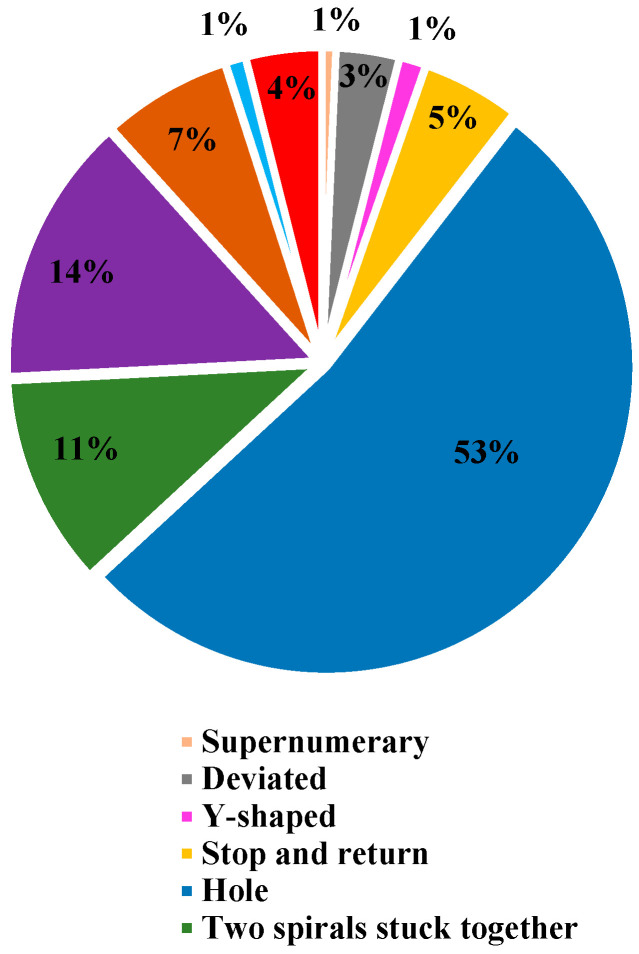
A pie graph representing the proportion of anomalies found in the orb webs of the spider *N. vigilans*. Among the 14,369 anomalies (*n* = 105), the highest percentage is hole (53%), while the lowest percentages are supernumerary (1%), Y-shaped (1%), and non-parallel (1%).

There were 7567 holes, the largest number of anomalies found in 105 webs. This accounts for 52.7% of all anomalies (Figure 5). Having more than two spirals stuck together (2026) was the second most abundant anomaly in 104 webs, accounting for 14.1% of all anomalies. Among all anomalies, 55 webs that had the lowest number of supernumeraries (100) accounted for 0.7% of the total anomalies (Table 1).

### 3.2. Relationship between Spider Body Measurements and Web Properties

There was a strong association between web characteristics and spider body characteristics. Vertical and horizontal web diameters, mesh size, capture area, CTL, and web size have significant relationships with spider body length, carapace width, and fourth leg length. Upper radii have a positive association with spider body length while showing a negative association with carapace width and fourth leg length. Upper spirals have a negative association with carapace width, while anchor points show a positive association with spider body length and carapace width (Table 2).

### 3.3. Relationship between Web Characteristics and Distance from the Road

There was a strong relationship between the number of entangled prey, plant height, and foliage radius with distance from the road, while there was a negative association between anchor points and web height from the ground (Table 3).

### 3.4. Relationship between Web Anomalies and Spider Body Measures

A negative association of the deviated anomaly of radii with spider body length and fourth leg length and more than two spirals stuck together also had a negative association with fourth leg length. In contrast, all other anomalies have a non-significant association with spider characteristics (Table 4).

### 3.5. Relationship between Web Anomalies and Distance from the Road

There was a significant correlation between anomalies and the distance from the road. The distance of webs from the road has led to a significant decrease in anomalies, such as deviations, y-shaped, holes, two spirals stuck together, discontinuities, non-parallel, and zigzag (α = 0.01).

## 4. Discussion

Atmospheric pollution harms human health and ecosystems. Traffic is the main source of environmental pollution. This research explored using spider webs as indicators of pollution. Spider webs proved to be effective indicators of road traffic emissions and might be even more reliable [41]. Areas near roads often experience frequent disturbances, such as noise and vibrations, which can cause physiological or behavioral changes in many animals [42]. Pollution, road disturbance, prey abundance, and diversity are also factors that influence the orb-web structure of *Neoscona vigilans* along roads. The spider web structure was also altered due to disruption [43], physiological changes [44], or changes in environmental conditions. Traffic disturbances cause stress in spiders, leading to physiological changes and altered behavior [45]. Different web characteristics and defects (anomalies) were observed and linked with disturbance along the road.

The spider webs built near roads were higher from the ground (Table 3), while webs built away from road had less height from the ground [46]. And more webs were discovered farther away from the road where the plant height and foliage radius were greater [47]. Spiders’ lifespan [48] and size [49] were shorter in the road vicinity [48], where the temperature was high due to traffic and greater diversity in older plots with more plant height and foliage cover [50]. Orb-weaving spiders seem to be less common near busy roads [51].

The spider body characteristics and web characteristics (Table 2) were strongly associated [52], which was also observed in this study when compared to distance from road disturbance, as larger spiders with more accurate webs were observed as distance from road disturbance increased (Table 3).

Spiders of smaller sizes constructed less sticky webs that were less successful at entangling prey, whereas larger size spiders weaved larger-size webs that entrapped a higher number of prey (*p* = 0.046) [53]. The humidity during web development influenced the size and stickiness of sticky spirals, which were necessary for capturing prey [54]. Traffic and exhaust emissions raised the temperature around roadways, causing humidity to drop and, as a result, reducing the size and capture area of the web [55]. When humidity was low, spiders constructed smaller webs with a more hygroscopic coating on sticky spirals. As spiders got older, they lost the hygroscopic material, causing their spirals to become less sticky, reducing the effectiveness of their webs in entangling prey and making them more vulnerable to rain and wind forces [56]. There was a small but significant negative correlation between road traffic and spider numbers [42].

Mesh size and web size were the two most important factors influencing the performance of webs [57]. A spider’s body length [58], fourth leg length [59], and carapace width [60] all had a positive relationship (*p* < 0.001) with its web capture area (Table 2). Spiders with longer body length had more upper radii, but spiders with larger carapace width and fourth leg length had fewer upper radii. Upper spirals had a strong negative association with carapace width and spider body length [61]. The radii were more in the lower half of the web [57], which had no association with spider body measurements. The fine size of spider silk had an impact on the physics and engineering of the web. Sticky spirals immobilized insects as they stroked the webs; alternatively, they absorbed the prey’s kinetic energy through the web’s radii, making the web stronger, more flexible, and more efficient [62]. Spiders made webs with larger radii and sticky spirals to absorb the kinetic energy generated by contact with large, fast-flying insects [63].

The vertical asymmetry in webs of *N. vigilans* aided spiders in finding food by allowing them to run downward faster [64]. This contradicted previous findings, which state that there is no association between spider traits and web asymmetry [65]. Because orb web-weaving spiders use gravity as a compass to create their webs, asymmetry in spider webs had no significant correlation with spider traits [66].

Carapace width was positively associated with mesh size [60] and CTL [67] (*p* < 0.001, *p* = 0.012). The spider’s web mesh size changed depending on how it caught its prey (63). Spiders with smaller legs were better able to entangle smaller prey in their webs [60]. On the other hand, webs with larger mesh sizes became less visible and trapped heavier prey [68], but this was rarely tested in the field [69]. Webs with smaller mesh sizes contained dense, sticky spirals that entangled more prey [70]. However, transmitting vehicle light during night made webs more visible to prey, and so prey avoided them [20]. As a result, webs closer to the road had less prey trapped (*p* = 0.020) due to heavy headlights. The farther away from the road, the more prey were caught in the webs.

During web creation, spiders utilized local knowledge to develop sticky spirals, which were guided by auxiliary spirals and previously placed capture spiral turns [71]. While spiders used past pieces to build new ones, mistakes sometimes happened and altered the structure and functionality of the web [20]. Defects in weaving behavior caused anomalies in the web structure. A strong association existed between the number of abnormalities per web and the length of the spider’s body and fourth leg (Table 4) [27,29]. There was a strong association between spider body size and leg dimensions, but deviation, hole, and abnormalities caused by displacement of leg position all showed a negative link with spider body length and fourth leg length [25,72]. Web building relied on spider legs, which contained muscles that were tired easily [73]. In the early stages of their decline in health, short-lived spiders exhibited many anomalies [74]. Several biotic and abiotic variables contributed to these differences [75]. Anomalies in web design were caused by the pollution produced by vehicles, some affected as with the usage of various pesticides in various fields, which altered the regular web architecture [76]. Because orb weavers constructed fresh webs each night, they were able to absorb more contaminants during web recycling, which was especially important beside roadways, where pollution levels are high [77,78]. Because of age-related declines in motor neuron coordination and other neurotoxins’ impacts on normal building behavior, road disturbance had a deleterious impact on web construction [79,80].

In order to find prey, web-building spiders relied on vibrations in the air and in their webs rather than their excellent vision [81]. In the end, spider web-building activity was impacted by various factors, including increased disturbance and pressure on web-anchor threads [82]. The webs near roads had more anchor threads because spiders experienced higher levels of noise pressure in close proximity to roads [83].

**Figure 6:**
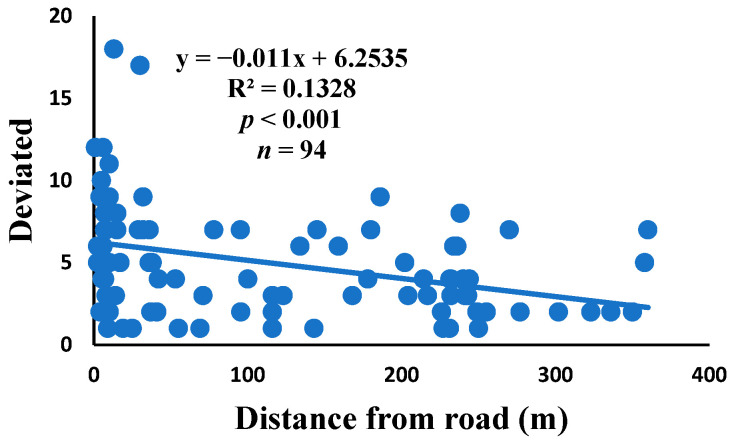
Correlation of deviated with distance.

**Figure 7:**
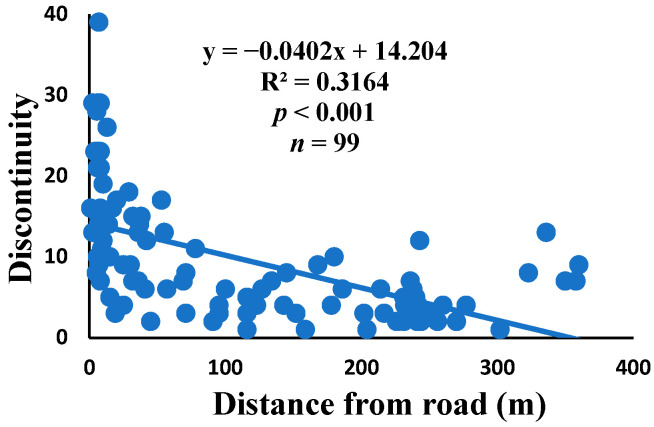
Correlation of discontinuity with distance.

**Figure 8:**
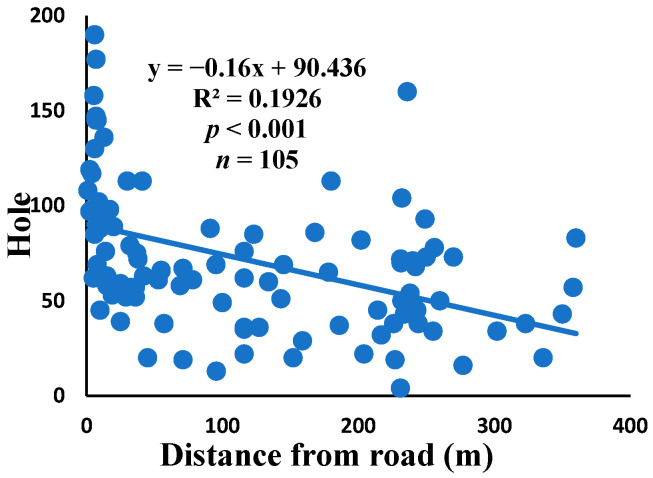
Correlation of hole with distance.

**Figure 9:**
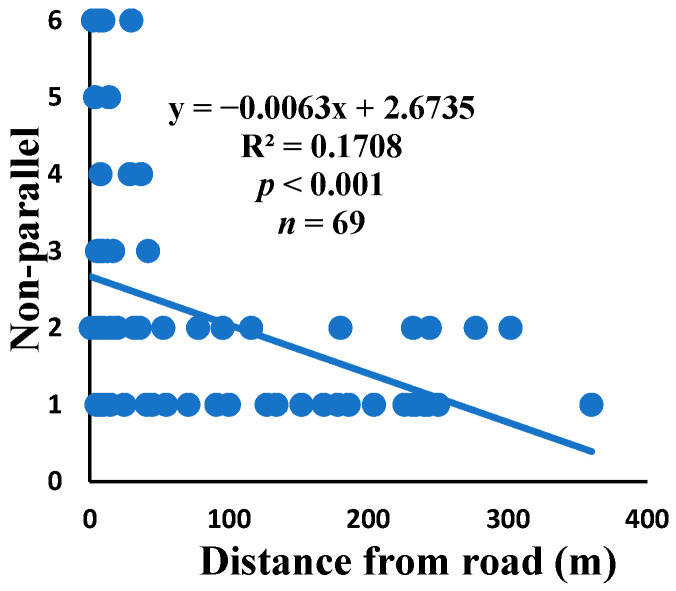
Correlation of non-parallel with distance.

**Figure 10:**
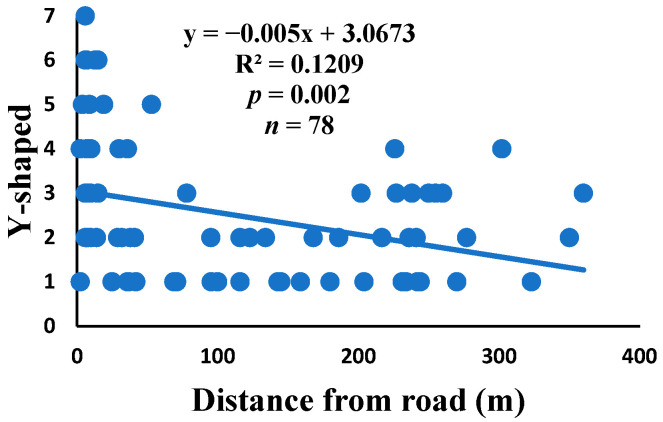
Correlation of y-shaped with distance.

**Figure 11:**
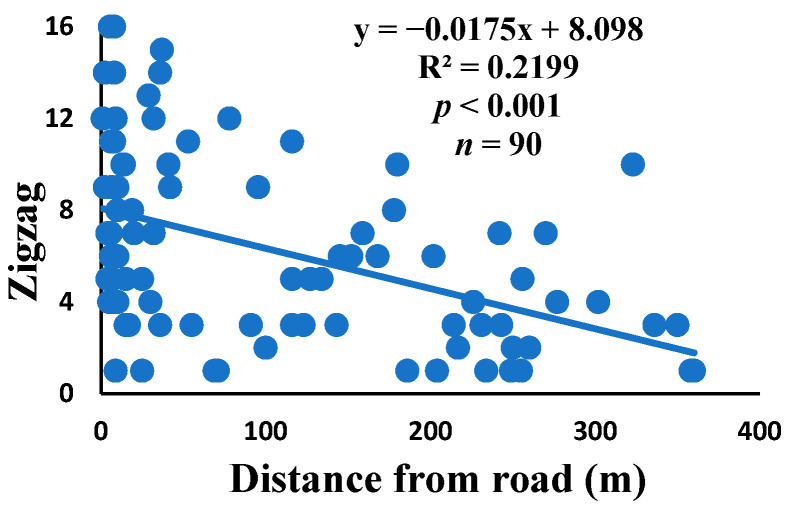
Correlation of zigzag with distance.

**Figure 12:**
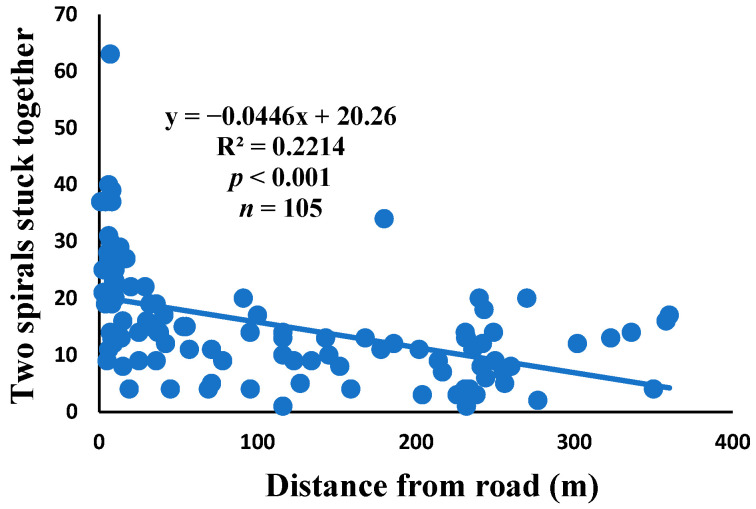
Correlation of two spirals stuck together with distance.

Spiders preferred appropriate locations to construct their webs based on the available space [84]. Webs constructed in their native habitats were found to exhibit imperfections and significant damage, affecting their ability to capture prey [85]. When behavior is linked to other traits, conflicting selection pressures can prevent adaptation, causing a mismatch between the actual and ideal behaviors in a new environment [86]. The creation of spider webs was altered by spiders in response to variations in the surrounding temperature [87]. The presence of a significant disturbance, heightened levels of pollutants, temperature, and pressure along the road had an impact on the creation of webs under windy conditions, which differs from webs created under quiet conditions [55]. Webs located in close proximity to the road exhibited a higher degree of damage and imperfections. A bigger mesh size of webs referred to the situation as the distance between radii increased, particularly on the periphery. In order to reduce the length of the radii, certain spiders employed the addition of subsidiary radii (supernumerary) that do not begin from the hub but rather from a more distant location [88]. This phenomenon had a negligible correlation with the distance from the road. When the damage in the web increased, the damping, stiffness, natural frequency, and transmissibility decreased [89]. The presence of disturbances along roadways leads to increased tensions in spider webs, resulting in the occurrence of additional anomalies (Figure 6, Figure 7, Figure 8, Figure 9, Figure 10, Figure 11 and Figure 12). Few webs had double-strand radii in the periphery, which formed a y-shaped anomaly in the high-tension region. In contrast, the low-tension region (hub) had single-strand radii [90]. A greater number of holes were found in spider webs along the road due to the increased activity of prey at high temperatures, which leads to flaws or damage in webs [91]. However, these holes reduced dramatically as the distance from the road increased. Pollution on the road caused motor neuron coordination failure in spiders, similar to the effects of many drugs. This was because the fractal dynamics of brain impulses created fractal webs [92].

Spiders play a crucial role as biological control agents, so it is necessary to take measures to ensure their survival if we want to keep ecosystems healthy. Pollution and disturbance disturb the typical behavior of many arthropods, including the orb-weaving spider, *N. vigilans*. The immediate abolition of all synthetic pesticides used in agriculture and other land management practices is critical. Automobiles and other human-caused hazards could be lessened with these steps taken. Anthropogenic activities, different environmental pollutants, and the level of disturbance can all be monitored using this research strategy.

## 5. Conclusions

Changes in the internal and external environments impact web construction. A positive correlation exists between spider body length, fourth leg length, carapace width, capture thread length, mesh size, web size, capture area, and vertical and horizontal web diameter and web size. There is a negative correlation between carapace width and upper spirals and radii, a positive association between radii and spider body length, and a negative association between radii and spider leg length. As one moves farther from the road, one notices a marked decline in web height from the ground and anchor points and an increase in the amount of prey and foliage radius. When two or more spirals are hooked together, the spider’s fourth leg length decreases, and when the spider deviates, the lengths of its body and legs both decrease. The behavior of *N. vigilans* is impacted by road disruption, pollution, and human activity. As one moves away from the road, the abnormalities in their normally occurring web properties diminish considerably. Road disturbance and other human-introduced activities negatively impact the normal behavior of living organisms. There is a need to overcome the challenges of increasing traffic flow to reduce disturbance and carbon emissions for the conservation and sustainability of the ecosystem. In the future, there is also a need for investigations of the long-term consequences of the disturbances in natural habitats and how different levels of pollution intensity or exposure duration affect web-building behavior and identifying their mechanisms of action.

## Figures and Tables

**Table 1:** The different anomalies were recorded in *N. vigilans’* webs. The relative percentage verss of the number of anomalies (the proportion of webs containing a specific form of anomaly and the mean number of anomalies per web) were computed using data collected from 105 webs originating from natural environments.

Anomalies	Percentage of Webs in Which an Anomaly Was Present	Percentage of Anomalies	The Average Number of Anomalies per Web	Total Anomalies	*n*
Hole	100%	52.7%	72.1	7567	105
Discontinuity	94.3%	6.8%	9.8	970	99
Supernumerary	52.4%	0.7%	1.8	100	55
Two spirals stuck together	100%	11.1%	15.1	1590	105
Nonparallel	65.7%	1%	2.2	149	69
Deviated	89.5%	3.3%	5.0	470	94
Zigzag	85.7%	3.9%	6.3	564	90
Y-shaped	74.3%	1.4%	2.5	196	78
More than two spirals stuck together	99%	14.1%	19.5	2026	104
Stop and return	99%	5.1%	7.1	737	104
Total		100%		14,369	

**Table 2:** Different body characteristics of spiders were measured, and the relationship was recorded with their web characteristics (*n* = 104, α = 0.01).

Web Characteristic	Fourth Leg Length (mm)		Carapace Width (mm)		Body Length (mm)	
	R^2^	*p*	R^2^	*p*	R^2^	*p*
Horizontal web diameter (cm)	0.145	<0.001 *	0.086	0.002	0.120	<0.001 *
Mesh size (mm)	0.279	<0.001 *	0.237	<0.001 *	0.181	<0.001 *
Upper radii	0.052	0.019	0.049	0.024	0.044	0.032
Anchor points	0.022	0.136	0.048	0.026	0.044	0.032
Capture area (cm^2^)	0.347	<0.001 *	0.241	<0.001 *	0.249	<0.001 *
Lower radii	0.030	0.077	0.030	0.081	0.026	0.104
Web height from ground (cm)	0.002	0.620	0.010	0.319	0.007	0.388
CTL (cm)	0.096	0.001 *	0.061	0.012	0.084	0.003
Upper spirals	0.035	0.059	0.045	0.031	0.019	0.158
No. of prey	0.038	0.048	0.036	0.055	0.039	0.046
Asymmetry	0.029	0.083	0.034	0.062	0.026	0.103
Lower spirals	0.003	0.562	0.007	0.404	0.001	0.784
Plant height (cm)	0.000	0.931	0.011	0.287	0.011	0.294
Web size (cm)	0.345	<0.001 *	0.247	<0.001 *	0.268	<0.001 *
Vertical web diameter (cm)	0.382	<0.001 *	0.295	<0.001 *	0.288	<0.001 *
Foliage radius (cm)	0.001	0.792	0.024	0.120	0.011	0.281

* significant association.

**Table 3:** Spider web characteristics were recorded, and the relationship with the distance of webs from the road was recorded (α = 0.01).

Web Characteristic	R^2^	*p*	*n*
Horizontal web diameter (cm)	0.006	0.181	105
Mesh size (mm)	0.025	0.632	105
Upper radii	0.021	0.268	105
Anchor points	0.066	0.001 *	105
Capture area (cm^2^)	0.027	0.165	105
Lower radii	0.006	0.865	105
Web height from ground (cm)	0.041	0.004 *	105
CTL (cm)	0.005	0.331	105
Upper spirals	0.000	0.771	105
No. of prey	0.093	0.020 *	94
Asymmetry	0.001	0.303	105
Lower spirals	0.001	0.520	105
Plant height (cm)	0.058	<0.001 *	105
Web size (cm)	0.028	0.149	105
Vertical web diameter (cm)	0.040	0.254	105
Foliage radius (cm)	0.060	0.001 *	105

* significant association.

**Table 4:** Different web anomalies were recorded, and their relationship with spider body characteristics was recorded (α = 0.01).

Web Anomaly	Fourth Leg Length (mm)		Carapace Width (mm)		Body Length (mm)		n
	R^2^	*p*	R^2^	*P*	R^2^	*p*	
Two spirals stuck together	0.001	0.793	0.012	0.269	0.007	0.382	104
Supernumerary	0.003	0.670	0.003	0.696	0.001	0.788	55
More than two spirals stuck together	0.057	0.014	0.028	0.091	0.031	0.076	104
Deviated	0.185	0.001 *	0.011	0.136	0.184	0.001 *	94
Discontinuity	0.020	0.159	0.001	0.794	0.010	0.316	99
Y-shaped	0.023	0.190	0.018	0.244	0.016	0.263	78
Non-parallel	0.000	0.906	0.007	0.493	0.001	0.761	69
Stop and return	0.000	0.936	0.000	0.907	0.000	0.971	104
Zigzag	0.000	0.994	0.003	0.632	0.007	0.443	90
Hole	0.015	0.209	0.001	0.819	0.003	0.570	104

* significant association.

## Data Availability

The original contributions presented in the study are included in the article, further inquiries can be directed to the corresponding author.
